# Top-Down CMOS-NEMS Polysilicon Nanowire with Piezoresistive Transduction

**DOI:** 10.3390/s150717036

**Published:** 2015-07-14

**Authors:** Eloi Marigó, Marc Sansa, Francesc Pérez-Murano, Arantxa Uranga, Núria Barniol

**Affiliations:** 1 Department of Electronics Engineering, Universitat Autònoma de Barcelona (UAB), Barcelona 08193, Spain; E-Mails: eloi_marigo@silterra.com (E.M.); arantxa.uranga@uab.es (A.U.); 2 Instituto de Microelectrónica de Barcelona (IMB-CNM-CSIC), Campus UAB, Barcelona 08193, Spain; E-Mails: marc.sansaperna@cea.fr (M.S.); francesc.perez@csic.es (F.P.-M.)

**Keywords:** NEMS, CMOS-NEMS, mechanical resonators, piezoresistive transduction, polysilicon nanowires

## Abstract

A top-down clamped-clamped beam integrated in a CMOS technology with a cross section of 500 nm × 280 nm has been electrostatic actuated and sensed using two different transduction methods: capacitive and piezoresistive. The resonator made from a single polysilicon layer has a fundamental in-plane resonance at 27 MHz. Piezoresistive transduction avoids the effect of the parasitic capacitance assessing the capability to use it and enhance the CMOS-NEMS resonators towards more efficient oscillator. The displacement derived from the capacitive transduction allows to compute the gauge factor for the polysilicon material available in the CMOS technology.

## 1. Introduction

The field of micro/nanoelectromechanical systems (MEMS/NEMS) is increasing its presence in many application areas because the advantages that they offer in terms of enhanced portability, reduced power consumption and reduced cost. The expected market for these devices is growing in the sensor field and also in signal processing for communications systems. Simple device structures like cantilevers and double clamped beams are used as building blocks in microsystems for a wide range of sensing applications [1,2,3,4,5]. Cantilevers and double clamped beams of nanometer scale dimensions improve the performance of sensors because of their enhanced sensitivity and higher resonance frequency. However, the lack of a high yield, high throughput fabrication method for NEMS prevents its industrial development. Recently, new approaches, like the so-called NEMS-Very Large Scale Integration (VLSI) have arisen enhancing the manufacturability capabilities and offering possibilities for massive manufacturing [6]. Regarding the development of NEMS for communications systems, one of the key applications is the possibility to substitute the quartz crystal with miniaturized mechanical resonators. As highlighted by the semiconductor industries [7] the achievement of frequency reference systems monolithically integrated in Complementary Metal Oxide Semiconductor (CMOS) processes, would be a major breakthrough towards a production-enabling technology.

Outstanding MEMS/NEMS performance can be achieved through the synergy with microelectronics. On the one hand, microelectronics technology enables the scaling down of dimensions by using advanced processing methods, like deep ultraviolet (UV) optical lithography. In addition integration of NEMS devices with integrated electronic circuits provides additional functionality, signal conditioning and better energy management.

To achieve an integrated system composed of NEMS devices and electronic circuits it is necessary that the mechanical movement be transduced into an electrical signal. However, the dimensional scaling down of MEMS devices makes an efficient motional transduction to the electrical domain challenging. Among the transduction methods suitable for monolithic integration, capacitive sensing is the most used. Unfortunately, scaling down capacitive transduced MEMS resonators provides huge motional impedances making very challenging the monolithical integration of oscillators or self-actuated systems [8]. Additionally capacitive NEMS produces high impedance mismatch (losses) in the case of RF systems. Despite these drawbacks, frequency oscillators fully integrated in CMOS circuits have been reported [9,10], although they are power demanding due to the high transimpedance gain needed to compensate for the high motional resistance. Some efforts to decrease this motional resistance by gap reduction or enhanced quality factor resonators have been proposed [11,12,13,14], but no one presents a substantial improvement in terms of reduced motional resistance at low bias voltage with scalable dimensions in CMOS technologies.

As an alternative to capacitive transduction, piezoresistive transduction has been proposed in order to decrease the motional impedance in MEMS resonators [15,16,17,18]. Piezoresistive transduction is a good integrable solution because incorporation of an integrated piezoresistance is compatible with preserving the small dimensions of nanomechanical resonators and the interface with the electronic circuits is simple. Some approaches dealing with piezoresistance transduction for MEMS resonators in CMOS technologies have been reported [12,18,19]. Zalalutdinov *et al.* [12] reported piezoresistive transduced CMOS-MEMS resonators with thermoelastic actuation. However their technological approach is based on the use of two polysilicon layers which are not available in CMOS technology nodes below 0.35 µm. Li *et al.* in [18] developed large metal-oxide stacked resonators with a bottom polysilicon layer acting as a piezoresistance requiring actuation voltages bigger than 100 V, which are not desired for a fully integrable CMOS-MEMS system. Finally Arcamone *et al.* in [19] required a dedicated process consisting of a pre-definition of the MEMS resonator on a Silicon-on-Insulator (SOI) wafer prior to the CMOS fabrication.

In this paper we present a capacitively actuated and piezoresistively transduced polysilicon double-clamped beam resonator fabricated and monolithically integrated in a commercial CMOS technology. It presents two main advantages in comparison with previous examples: (a) smaller dimensions (the beam dimensions are 500 nm width and 282 nm thick); and (b) the entire body of the resonator is used as a piezoresistor. Electrical measurements demonstrate that it is feasible to use piezoresistive transduction in nanometer scale mechanical resonators fabricated using non-modified commercial CMOS technologies. Comparing the response of the same device for capacitive transduction and piezoresistive transduction allows to establish the material properties (*i.e.* gauge factor for the integrated polysilicon layer).

## 2. Experimental Section

The clamped-clamped beam (CC-beam) is fabricated in a 0.35 µm CMOS technology from AMS (Austria Microsystems, Graz, Austria). This technology is based on two poly-silicon layers and four metals. The beam resonator is defined on the poly1 layer (thickness of 282 nm) using the silicon dioxide as the sacrificial layer.

**Figure 1:**
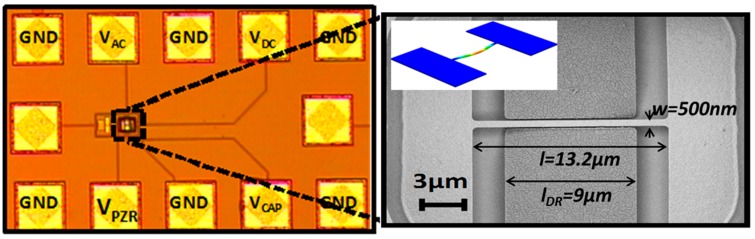
**Left**: Optical image of the integrated Polysilicon clamped-clamped beam in the CMOS technology; **Right**: Detail of the polysilicon resonator after its releasing in a SEM image. The thickness of the double clamped-beam is 282 nm and the gaps between driver electrodes and beam are 100 nm. The inset shows Coventor simulations for the first in plane resonant mode at f_0_ = 25.5 MHz.

The electrostatic actuation for the resonant NEMS operation is performed through the fixed polysilicon electrode (from the poly2 layer) placed 100 nm besides the CC-beam (in-plane actuation and movement, see Figure 1). Efficient vertical alignment between the two polysilicon layers for an in-plane movement is obtained due to their different thicknesses (280 nm for poly1 and 200 nm for poly2), the insulator layer thickness between them (40 nm) and the conformal deposition used [20,21]. The capacitive sensing is done by an additional driver of poly2 at the other side of the beam (in a two-port symmetrical configuration). Equal spaced driver electrodes are used. For piezoresistive sensing the two anchors of the beam are connected to pads to allow current flowing through the resonator.

The CC beam resonator is fabricated by the CMOS foundry following its standard processes. As a special requirement two square vias and an opening pad are defined above the resonator to allow the post CMOS releasing process for the NEMS resonator which will be done in-house. This process is a maskless wet etching of the silicon dioxide around the CC-beam using a buffered HF acid solution [20]. An optical image of the CMOS-NEMS CC-beam showing the pads for the electrical characterization and SEM image of the fabricated device is shown in Figure 1.

According to the technological specifications, the sheet resistance of the polysilicon layers is in the range 5–11 Ω/square and the maximum current density that can sustain is 0.5 mA/µm. In order to keep the resistance low, the width of the CC-beam is set at 0.5 µm, larger than the minimum allowed in the technology. The length of the beam is fixed to 13.2 µm. With these dimensions the maximum dc current allowed is 250 µA while the total resistance of the beam is in the range between 132 Ω and 290 Ω. The theoretical resonant frequency for the fundamental lateral in-plane mode is 25.2 MHz (assuming a polysilicon Young’s modulus of 169 GPa and a mass density of 2330 kg/m^3^).

The CC beam is electrostatically actuated applying an AC signal, v*_ac_ = V_ac1_* sin*wt*, at the capacitor defined by the coupling area between one of the driver electrodes and the CC-beam (C_o_ in equilibrium), while the beam is biased with a DC voltage, V_DC_. Considering small displacements (x) in comparison with the gap (g) and assuming a parallel plate capacitor approximation, $C_{act}=C_{0}\frac{g}{g+x}$, the electrical force component at the resonance frequency of the beam due to this electrostatic excitation is:

(1) $$F_{x}=-\frac{C_{0}}{g}V_{DC}\cdot V_{ac1}\sin(wt)$$

Assuming a simple harmonic oscillator with a quality factor *Q*, and elastic constant *k*, the maximum displacement of the beam in the *x* direction at its resonance frequency can be computed as a function of the actuation voltages according to Equation (2):

(2) $$x_{\max}=\frac{QF_{x}}{k}=\frac{Q}{k}\frac{C_{0}}{g}V_{DC}V_{ac1}$$

### 2.1. Capacitive Sensing

The motion of the beam, due to the AC actuation in the excitation driver, produces changes in the readout capacitor between the CC-beam and the read-out driver, C_r_, which induces a current in this output electrode:

(3) $$I_{cap}=\frac{\partial \left(V_{DC}\cdot C_{r}\right)}{\partial t}=C_{r}\frac{\partial V_{DC}}{\partial t}+V_{DC}\frac{\partial C_{r}}{\partial t}=V_{DC}\frac{\partial C_{r}}{\partial t}$$

The previous Equation could be expressed in terms of velocity of the displacement:

(4) $$I_{cap}=V_{DC}\frac{\partial C_{r}}{\partial x}\frac{\partial x}{\partial t}=V_{DC}\frac{\partial }{\partial x}\left(C_{0}\frac{g}{g-x}\right)\frac{\partial x}{\partial t}\approx V_{DC}\frac{C_{0}}{g}\frac{\partial x}{\partial t}$$

for small displacements (x << g). Finally and considering a simple harmonic oscillator, with sinusoidal displacement at the resonance frequency, $f_{RF}$, $x(t)=x_{\max}\sin(2\pi f_{RF}t)$, the maximum motional current at the resonance frequency can be written in terms of the maximum displacement due to the electrostatic actuation force, Equation (5):

(5) $$I_{cap\max}\approx V_{DC}\frac{C_{0}}{g}2\pi f_{RF}x_{\max}$$

### 2.2. Piezoresistive Sensing

Piezoresistive effect is based on the change of the resistance of the resonator as a function of shape deformation or strain, *ε_l_ = Δl/l*, where *G* represents the gauge factor and ΔR/R_0_ is the relative change in the specific resistance:

(6) $$\frac{\Delta R}{R_{0}}=G\cdot \varepsilon_{l}$$

considering that for the CC-beam structure the main contribution is the longitudinal strain due to bending moment. In a symmetric beam equal tensile and compressive strains distributed on the opposite sides of the beam will produce zero change in total resistance. However, the resonant movement of the beam produces a change on its length which it turns out to a non-negligible longitudinal strain through the CC-beam. This strain will be the responsible for a change in resistance and thus for the piezoresistive transduction in the symmetric CC-beam. To compute the total lengthening of the beam due to the movement, *l_elongated_*, the simple arch length model is used ($\Delta l^{2}=\Delta x^{2}+\Delta y^{2}$):

(7) $$l_{elongated}=\int_{0}^{l}dl=\int_{0}^{l}\sqrt{{\left(dy\right)}^{2}+{\left(dx\right)}^{2}}=\int_{0}^{l}(\sqrt{{\left(\frac{dx}{dy}\right)}^{2}+1})dy\approx \int_{0}^{l}\left[1+\frac{1}{2}{\left(\frac{dx}{dy}\right)}^{2}\right]dy$$

The longitudinal strain becomes:

(8) $$\varepsilon_{l}=\frac{\Delta l}{l}=\frac{l_{elongated}-l}{l}\approx \frac{1}{2l}\int_{0}^{l}{\left(\frac{dx}{dy}\right)}^{2}dy=\frac{\pi^{2}}{4}\frac{x_{\max}^{2}(t)}{l^{2}}$$

where it has been assumed $x(y,t)=x_{\max}(t)\sin\frac{\pi y}{l}$, as the displacement for the fundamental in-plane resonant mode for the CC-beam and *x_ma_*_x_(t) the displacement at the beam center. Finally the change in resistance could be expressed as:

(9) $$\frac{\Delta R}{R_{0}}=G\cdot \frac{\pi^{2}}{4}{\left(\frac{x_{\max}(t)}{l}\right)}^{2}$$

According to Equation (9), the maximum change in resistance depends upon the square of displacement and thus the signal will be at twice the beam resonance frequency (or similarly the resistance is changing twice for each period of the resonance frequency). Finally the output current is measured applying a voltage signal over the resonator, V_CCB_:

(10) $$I_{piezo}\pm \Delta I_{piezo}=\frac{V_{CCB}}{R_{0}\mp \Delta R}≅\frac{V_{CCB}}{R_{0}}(1\pm \frac{\Delta R}{R_{0}})$$

providing a transduction of the beam displacement assuming small variations *ΔR << R_0_* [16]. From this last expression the variation of current, ΔI_piezo_, which will be produced due to the change of resistance during the clamped-clamped beam movement can be related to the displacement according to next Equation (rearrangement terms in Equations (9) and (10)):

(11) $$\Delta I_{piezo}(t)=I_{piezo}\frac{\Delta R}{R_{0}}=\frac{V_{CCB}}{R_{0}}\left[G\cdot \frac{\pi^{2}}{4}{\left(\frac{x_{\max}(t)}{l}\right)}^{2}\right]$$

This principle has been successfully used and reported for silicon nanowire mechanical resonators thanks to the presence of an enhanced gauge factor [22,23]. In these works, a down-mixing scheme for the displacement transduction is used, taking profit of the quadratic dependence of the piezoresistive current on the displacement (Equation (11)). This down-mixing scheme is based on applying to the CC-beam a sinusoidal waveform of a frequency slightly different than the double of the beam resonance frequency ($V_{CCB}=V_{AC2}\cos2\pi (2f_{RF}+\Delta f)t$), at the same time that the beam is capacitively actuated at its resonance frequency. With this capacitive actuation the time dependent displacement at the beam center can be written as $x_{\max}(t)=x_{\max}\cos(2\pi f_{RF}t$). Consequently the transduced piezoresistive current corresponding to the displacement of the CC-beam has a frequency component at *Δf* which can be easily acquired using a lock-in instrument. Substituting these signals in Equation (11)*,* the piezoresistive current due to the resonant displacement of the beam can be written as Equation (12):

(12) $$\Delta I_{piezo}=\frac{1}{4}\frac{V_{AC2}}{R_{0}}\left[G\cdot \frac{\pi^{2}}{4}{\left(\frac{x_{\max}}{l}\right)}^{2}\right]$$

In this paper we have used the principle and transducing scheme explained above to characterize the piezoresistive transduction in nanomechanical resonators fabricated using a commercial CMOS technology and so, using the available layers of this CMOS technology.

## 3. Results and Discussion

### 3.1. Capacitive Transduction

**Figure 2:**
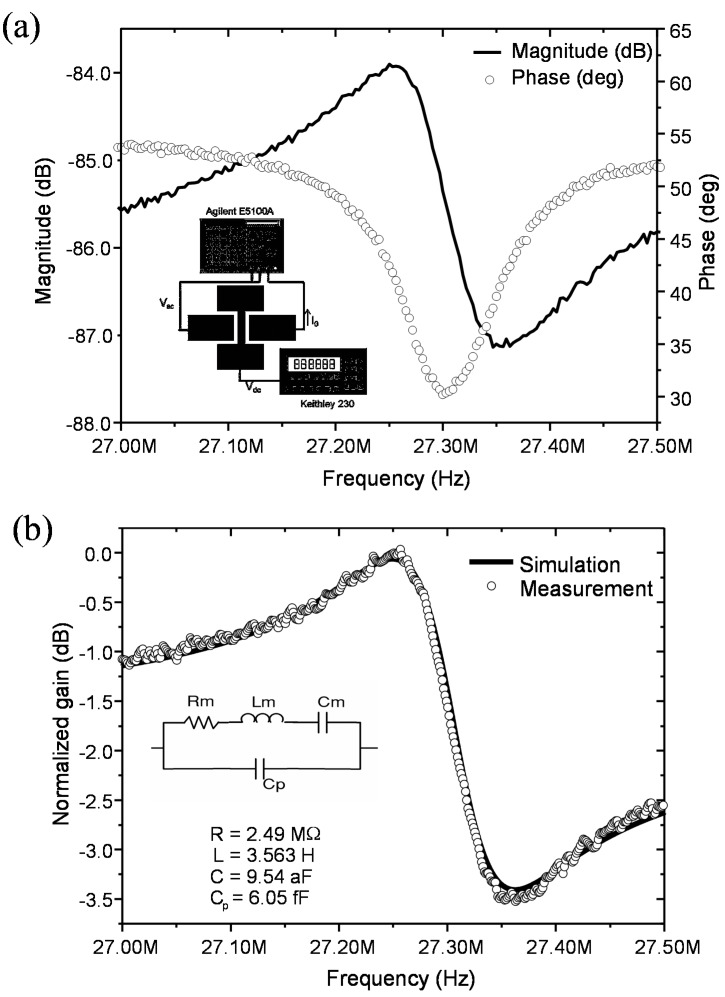
(**a**) Experimental frequency response (magnitude and phase) and Electrical characterization set-up for capacitive actuation and sensing; (**b**) Fitting of the experimental frequency response with the electrical equivalent circuit shown in the inset.

The frequency response of the CC beam using capacitive read-out is acquired directly from a network analyser according to the set-up of Figure 2a.

A two port configuration is used. Each driver electrode is used for the actuation and the read-out respectively. The CC beam is kept at a constant bias voltage. Applying an AC signal with a power of P = 10 dBm and Vdc = 15 V the magnitude and phase for the gain obtained from the network analyser are shown in Figure 2a. The frequency response in Figure 2a shows the resonance peak due to the mechanical oscillation/response of the device together with the anti-resonance electrical response due to the parasitic capacitance between the two drivers. In the simple linear electrical model for these mechanical resonators an RLC in parallel with C_p_ (see inset of Figure 2b) can be assumed. Figure 2b shows the fitting with an electrical RLC//C circuit of the experimental frequency response. According to the fitting, a motional resistance of 2.4 ΜΩ is obtained. The computed motional current at the resonance is 292 nA when an input power of 10 dBm is delivered to the NEMs by the network analyzer, (equivalent to 0.7 V rms assuming a 50 Ω load). In our setup the load in the network analyzer is in parallel with the reference input, thus even in the case of high input impedance from the NEMS, the equivalent load is close to the nominal 50 Ω. With this motional current we can compute the maximum beam displacement at the resonance frequency from Equation (5), obtaining a value of 52 nm, (with C_0_, the coupling capacitance between CC-beam and reading electrode, C_0_ = 0.22 fF, V_DC_ = 15 V, g = 100 nm and f_r_, the experimental resonance frequency f_r_ = 27 MHz). An almost equal value, 58 nm, is obtained from the computed electrostatic actuation force (Equation (2)) under the same conditions (A = 0.7 Vrms, V_DC_ = 15 V) and considering a quality factor, Q = 100 (extracted from the experimental frequency response of Figure 2) and a spring constant of k = 40 N/m, computed from finite model simulations (Coventor).

### 3.2. Piezoresistive Transduction

**Figure 3:**
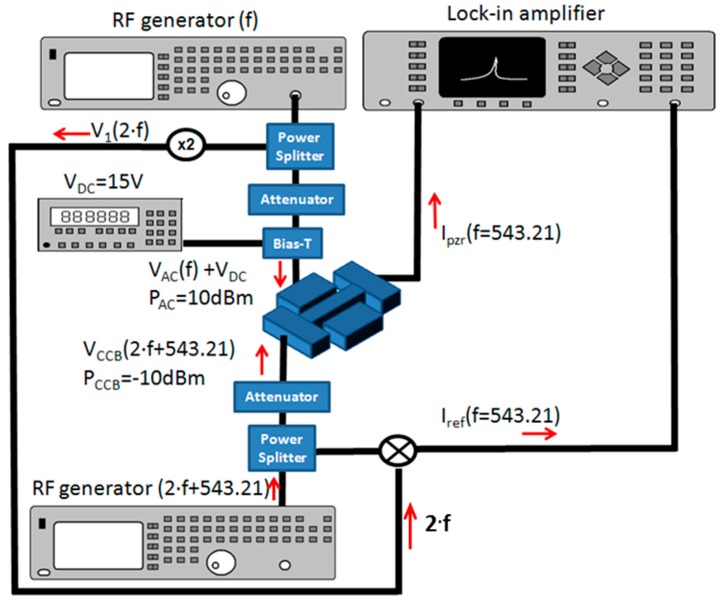
Piezoresistive sensing set-up with an electrostatic excitation. f will be around the first mode resonance frequency of the CMOS-NEMS CC-beam (around f = 27 MHz), and the low frequency is in our case 543 Hz.

The set-up for piezoresistive sensing is based on a downmixing scheme using the NEMS CC beam as a mixer in order to detect its motion at low frequencies (Figure 3). This technique often called two-source, double-frequency technique has been previously used to characterize bottom-up and also top-down crystalline silicon nanowires [23]. The actuation electrode is connected to an excitation signal with a frequency, f_RF_, which is equally to the first lateral mode of the CC-beam. This signal along with a bias voltage V_DC_, induces the motion of the beam producing its resonance at f_RF_. This beam motion produces a change in resistance which will be at 2f_RF_ due to the quadratic dependence of the resistance *versus* the displacement (piezoresistance effect, Equation (9)). In order to produce a down-mixing in the final piezoresistance current, an additional signal at 2f_RF_ + Δf is applied directly to the CC-beam. In this way a mixing process will take place at the CC-beam, producing a piezoresistive signal proportional to the product of the signals with frequencies 2f_RF_ and 2f_RF_ + Δf and thus composed of several harmonics, one of which is at Δf. Finally the lock-in amplifier will detect only the component at Δf, neglecting all the others. This reference signal for the lock-in is generated through a mixer and a frequency doubler. A lock-in amplifier is used instead of the network analyzer due to the benefits of using a superheterodyne receiver where a known low frequency reference signal is multiplied by the input signal and amplified, so the scheme is capable to detect small signals even buried in noise.

**Figure 4:**
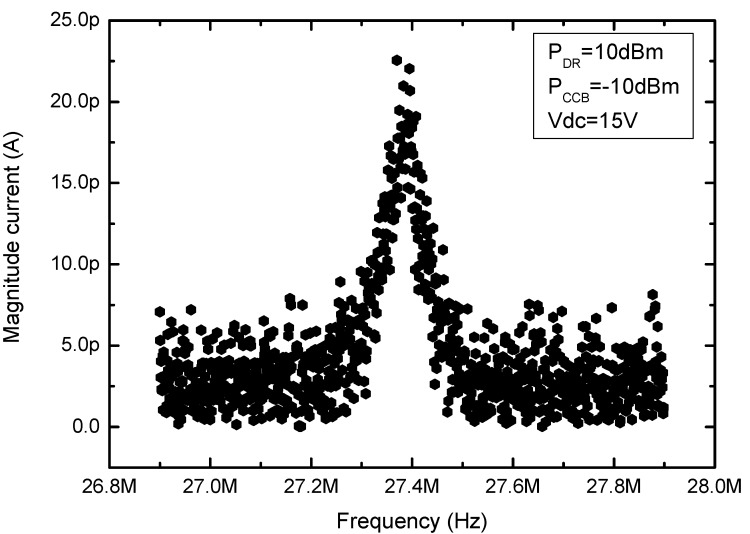
Frequency response obtained from the CMOS-NEMS clamped-clamped beam with the piezoresistive transduction method in vacuum.

It is important to consider that the density current flow through the clamped-clamped beam limits the maximum power applied over the resonator to prevent its melting. However the discrete mixer requires enough power to work properly, so a voltage controlled attenuator is placed between the power splitter and the resonator. With an AC signal power of 10 dBm the discrete mixer works properly and with an attenuation of 17 dB the resulting power applied directly to the resonator is −10 dBm. Maintaining a DC bias voltage of 15 V, an AC actuation voltage of 10 dBm and an AC voltage through the beam of −10 dBm, the motional current across the beam presents the frequency response depicted in Figure 4 (with a frequency offset, Δf = 543.21 Hz).

The obtained experimental frequency response clearly show the resonance peak which is produced by the piezoresistance change due to the beam elongation during resonant displacement.

Once proved the feasibility of piezoresistive transduction in a CMOS-NEMS beam considering only the strain produced due to the change in the beam length, some parameters from the resonator can be extracted. One of the key characteristics in order to establish the applicability of the piezoresistive sensing is the gauge factor. Note that the polysilicon layer is the standard layer in the CMOS technology for defining the gates of the MOS transistors. In order to extract the gauge factor we consider that the vibration amplitude of the clamped-clamped beam will be the same than the one obtained with the capacitive transduction. Although a mixing actuation is performed due to the two AC signals applied (the ac signal applied to the driver electrode, V_ac1_, and the AC signal directly applied to the beam, V_ac2_), the actuation force component at the resonance frequency of the beam will be the same than in the capacitive transduction case. In both cases the same actuation voltages, P = 10 dBm and V_DC_ = 15 V are used.

Taking into account an experimental piezoresistive peak current of 22 pA at resonance, a maximum vibration amplitude of 52 nm (extracted from the capacitive measurement under the same actuation electrostatic force); R_0_ = 2.75 kΩ (equivalent to the serial resistance between clamped-clamped beam, 250 Ω and the input resistance of the lock-in amplifier in low noise mode of 2.5 kΩ ); and V_AC2_ = 140 mV (corresponding to −10 dBm applied to a load impedance of 2.9 kΩ), a gauge factor of 0.05 is extracted from Equation (12). The gauge factor obtained is low if we compare with the values obtained for other piezoresistive resonators [8,12,15,16,17,18,19,22,23]. Only in [12,18] the polysilicon material from a CMOS technology is used, and in both cases the dimensions of the polysilicon structure are considerably larger. For instance, in [18] the polysilicon layer of a CMOS technology has been used as a simple resistance, constituting one of the building blocks of a metal-oxide stacked structure. In this case much bigger piezoresistance current through the polysilicon was allowed due to the bigger dimensions used contrary to the low current level allowed in our very small CC-beam resonator. Similarly larger piezoresistance coefficients were reported in the case of small nanowires [22,23] although in these cases crystalline silicon was the structural material instead of polysilicon. In [24] an in-depth study of the piezoresistive effect in top-down fabricated silicon nanowires (with nanowires from similar cross section than the one presented here) is made. One of the conclusions in [24] is that a much lower piezoresistance effect is computed for polysilicon nanowires in comparison with the crystalline silicon ones, emphasizing the dependence on the fabrication process. Despite of this, we have been capable to sense the piezoresistive current and successfully transduce the movement of the clamped-clamped polysilicon beam at resonance. Taking into account that the layer employed have been the original one used in the commercial CMOS process, we believe that there is room for improvement by introducing some modifications in the process that would improve the electromechanical properties of the polysilicon.

## 4. Conclusions

The dynamic displacement of a CMOS-NEMS clamped-clamped beam at resonance frequencies of 27 MHz has been successfully transduced capacitively as well as piezoresistively. From the measurements it has been possible to compute the gauge factor for the polysilicon material obtaining a lower value than expected. From these results it can be concluded that the piezoresistance transduction for top-down CMOS-NEMS polysilicon resonators is possible, but requires larger structures that can sustain larger deformations for an efficient transduction.
